# Pectus patient information website has improved access to care and patient reported outcomes

**DOI:** 10.1186/s13019-016-0470-7

**Published:** 2016-04-26

**Authors:** Theofano Tikka, Joanne Webb, Paula Agostini, Amy Kerr, Glenn Mannion, Richard S. Steyn, Ehab Bishay, Maninder S. Kalkat, Pala B Rajesh, Babu Naidu

**Affiliations:** Department of Thoracic Surgery, Heart of England NHS Foundation Trust, Bordesley Green East, Birmingham, UK; College of Medical and Dental Sciences, University of Birmingham, Edgbaston, Birmingham, UK

**Keywords:** Pectus website, Information

## Abstract

**Background:**

Pectus is the most common congenital disorder. Awareness amongst primary care physicians and the general public is poor. NHS commissioning bodies plan to withdraw funding for this surgery because they deem a lack of sufficient evidence of benefit. The purpose of this study is to assess the effects of introducing a patient information website on referral and activity patterns and on patients reported outcomes.

**Methods:**

We produced an innovative information website, www.pectus.co.uk, accessible to the general public, providing information about pectus deformities; management options and advice about surgery. Referral patterns and number of cases where studied before and after the introduction of the website in 2010. Patients’ satisfaction post-op was assessed using the Brompton’s single step questionnaire (SSQ).

**Results:**

The website had considerable traffic with 2179 hits in 2012, 4983 in 2013 and 7416 in 2014. This has led to 1421 contacts and 372 email enquiries. These emails have resulted in an increased number of patients who have been assessed and go on to have surgery. We asked 59 pectus excavatum patients who were operated from 2008 to 2014 to complete the SSQ. We received 32 replies. Eighty-four percent (16/19) of patients who visited the website and then underwent surgery, found the website useful. All patients scored satisfactorily in SSQ. Even though those who visited the website tended to be more satisfied with the surgical outcomes this did not reach statistical significance. This group of patients said that would have the operation again given the option compared to 76.9 % of the group who did not visit the website before surgery (*p*=0.031). Despite the fact that patients who visited the website experienced more post-operative complications were equally or more satisfied with post-operative outcomes. The overall SSQ obtainable score was not different for the two subgroups, being more widespread in the group that did not visit the website.

**Conclusions:**

The introduction of a pectus patient information website has significantly improved access to specialised services. Patients are overall highly satisfied with the surgical outcomes.

## Background

Pectus deformities affect one in every 400 children and young adults. It has a male predominance. There are two main type of pectus: pectus excavatum (PE) or funnel chest which is the commonest type and pectus carinatum (PC) or pigeon chest [1]. The negative psychosocial and physiological effects of pectus are widely accepted. Adolescents with pectus deformities tend to develop negative self-cognition as a results of health over-attention by themselves and their close relatives and friends. Altered body self-awareness can potentially results in behavioural problems, and build-up of negative emotions. The inter-link between psychosocial and physiological health can lead to an exacerbation of disease status in this group of young patients [2].

In the UK pectus correction is currently performed in NHS hospitals with an average of 400 operations performed every year. Nevertheless the NHS England plans to withdraw financial support for this operation. It is stated that there is currently ‘no sufficient evidence supporting routine commission of surgical correction of pectus deformities’ [3]. Adding to this, pectus awareness amongst health care physician and the general public is limited hence reducing the number of patients who get an appropriate treatment for their condition. Established psychosocial impairment is currently the main drive for a referral for surgical correction [4]. The negative psychosocial and physiological effects of pectus can be reversed with surgery and transform patients’ quality of life [5, 6]. Nuss and the modified Ravitch operation are the most common surgical treatment modalities [7, 8].

Over the last decade, internet resources have become a popular method of education for patients and their families. Studies have shown that patients seeking healthcare information on the internet have found it a useful resource which help them get a better understanding of their disease, the treatment options and prognosis [9]. The collected information can educate the general public but also enables patients to decide on the preferred treatment option for their pathology after discussion with their physician [10].

Two recurring themes were observed in patients who had pectus surgery in our department. Firstly, there was a lack of easily accessible information regarding their condition that therefore created a heightened level of anxiety and uncertainty. This also contributes to many general medical practitioners mis-diagnosing symptoms and being reluctant to refer patients to specialised thoracic units for further assessment of their condition. Pectus patients are often ‘fobbed off’ that their deformity is insignificant and their symptoms are dismissed from the primary care physicians [11, 12]. Therefore our thoracic surgery unit developed an innovative patient information website providing information about pectus [13].

In this paper we present the effect of the website on referral patterns in our unit and we assess patients’ satisfaction with the operation, the website and post-surgery outcomes.

## Methods

Our regional thoracic surgery unit developed a pectus information website, accessible to the general public that provides information about pectus deformities, management options, what is involved in having surgery and its benefits and risks. It also includes pre and post operation images and general advice about surgery and the recovery process in hospital and after discharge home. The website contains background information about pectus pathophysiology and manifestation.

The content of the website was formulated by a group of health care professionals including doctors; nurses; physiotherapists with the help of the medical illustration department. The website was refined after receiving feedback from patients who had already had surgery. The website went live in January 2010.

All e-mail enquiries generated from the website were answered by a single consultant (author BN) where possible dealing with enquiry and if appropriate signposting the enquirer to seek a referral by their GP to the local regional thoracic centre. In some cases this was our hospital. The number of hits on the website and e-mail enquiries were collected using google analytics carried out on the 4th of August 2015. The number of pectus surgery from 2007 to 2014 were identified from the prospectively collected unit surgical database that is verified prior to submission to the national database.

A prospective study was conducted to assess the effect of the launch of the website on referral patterns and patients satisfaction post-surgery. All patients who underwent primary Nuss operation in our institution from the launch of our website in January 2010 up to August 2014 were invited to participate. Data on the patient’s surgery; demographics and outcomes were collected from the prospective clinical database. Surgery selection was principally for cosmetic indications. Selection and management followed a standardised unit protocol.

The study was registered with the Birmingham Heartlands Hospital audit department. Patients were asked to answer an on-line questionnaire created for the purpose of the study. They were asked to comment on the website and the single step questionnaire (SSQ) was used to assess patients’ satisfaction post-surgery. It includes a total of 16 questions measuring physical and psychosocial effects of pectus surgery. These questions assess the degree of satisfaction by a single specific question but also by an overall obtainable score. The maximum possible score is 95, with a score of more than 41 being considered satisfactory [14].

Our cohort was sub-grouped into those who visited the website prior to pectus surgery and those who did not. Results are expressed as mean (SD or 95 % CI) for continuous variables and as a percentage for categorical variables. Univariate analysis of patients’ baseline characteristics was performed using Pearson’s chi-square test or Fisher’s exact test where appropriate for categorical variables and Mann Whitney U, Kruskal Wallis test or independent sample t test for continuous variables. The Mann Whitney U test was used to determine statistical significance of difference in the SSQ in our sub-groups. The IBM SPPS Statistics 20 was used to analyse the dataset.

## Results

The website had considerable traffic with 2179 hits in 2012, 4983 in 2013 and 7416 in 2014 with an average session duration of 3 min 18 s. This has led to 1421 contacts and 372 email enquiries. The website is registered with Google using a validated account, it contains unique meta/keywords and a multitude of inbound and outbound links to and from other sites.

There were 59 patients who had Nuss surgery for PE repair during the study period, of whom 32 replied to our on-line questionnaire. There were no differences in baseline characteristics between responders and non-responders (Table 1). Non-responders were excluded from any further analysis. Majority of responders were males (94 %). Their mean age was 20 (±5). Their overall mean hospital stay was 4.3 days (±1). A post-operative complication was observed in 3 patients (9 %). These were formation of haematoma requiring return to theatre for evacuation and arrest of bleeding; small apical pneumothorax with minor bar prominence treated conservatively and infected granulation tissue over wound site treated with oral antibiotics. A total of 15 patients had their bar removed by the time they completed the questionnaire (47 %). The mean time of bar removal was 29.4 months (95 % CI 26–32). On average patients filled the questionnaire 2.7 years after surgery (95 % CI 2.1–3.2).

**Table 1:** Baseline characteristics of those who responded and those who did not respond to our online questionnaire

Variable	Value	Responders (*n* = 32)	Non-responders (*n* = 27)	*p*-value
Age	Mean (95 % CI)	20 (18–22)	19 (17.7–20)	0.718
Gender	Male	30	27	0.495
	Female	2	0	
Length of hospital stay (days)	Mean (95 % CI)	4.3 (4–4.7)	4.5 (4–5)	0.981
Post-operative complications	Yes	3	3	0.579
	No	29	27	
Bar Removed	Yes	15	15	0.506
	No	17	12	

**Fig 1:**
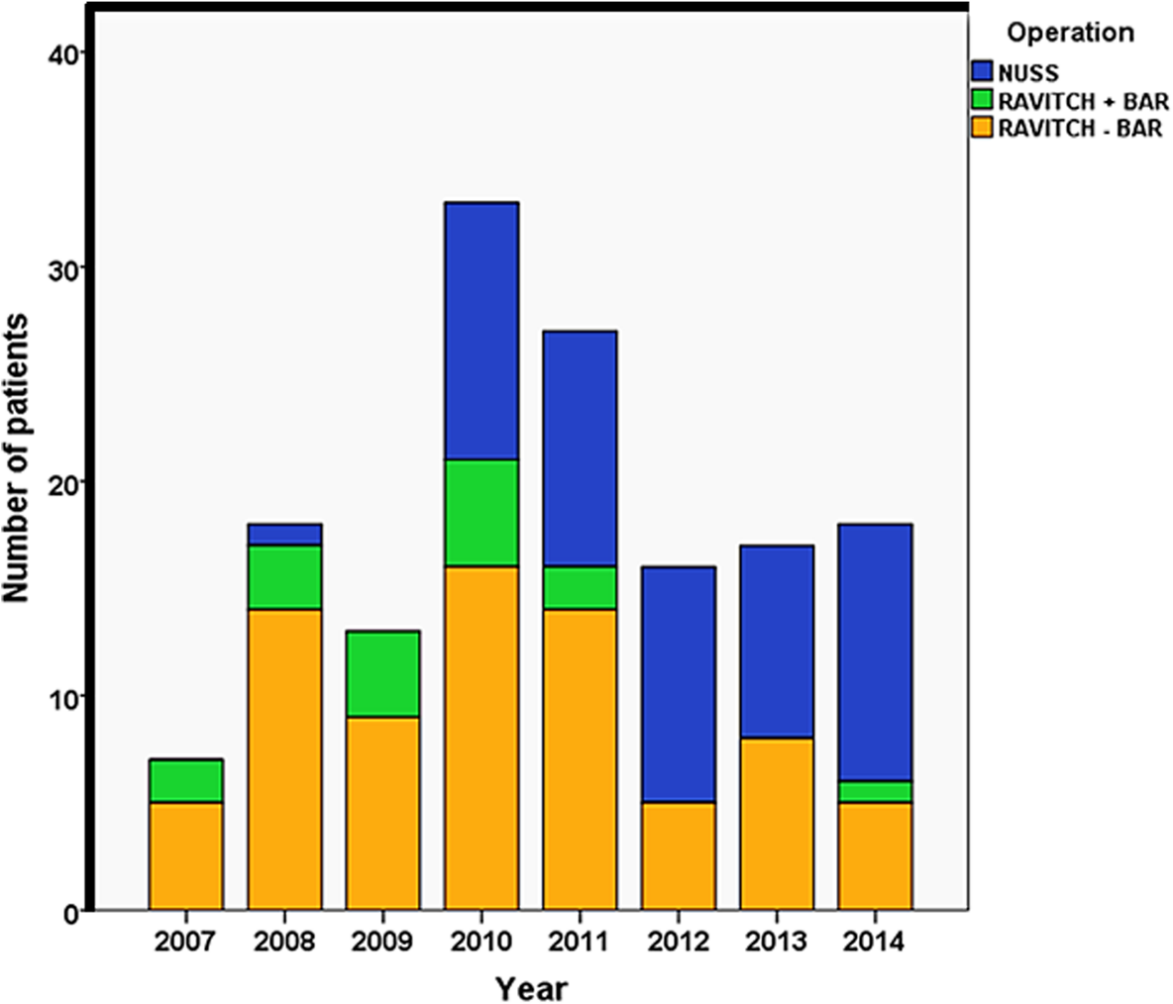
All patient who had pectus surgery in our department clustered by year of operation (from 2007 to August 2014)

From the 32 patients who answered our on-line questionnaire, 19 visited the website before having the surgery, 11 of those before being seen in our hospital for their initial assessment. The majority of them found the information on the website useful or very useful (*n* = 16, 84.2 %). Since the launch of the website there were an average 11 fold increase in the number of patients seen and treated for pectus in our department with the Nuss operation (Fig. 1).

We sub-grouped our cohort in 2 distinct groups: patients who visited the website prior to surgery and to those who did not. Patients in the two groups had the same baseline characteristics (Table 2). All three patients who developed a post-operative complication had visited the website prior to surgery.

**Table 2:** Baseline characteristics of patients visited and not visited the website prior to surgery, including univariate analysis

Variable	Value	Visited website (*n* = 19)	Did not visit website (*n* = 13)	*p*-value
Age	Mean (95 % CI)	19.9 (17.5–22.3)	20.3 (17–23.5)	0.821
Gender	Male	19	11	0.157
	Female	0	2	
Length of hospital stay (days)	Mean (95 % CI)	4.5 (3.9–4.9)	4.2 (3.8–4.6)	0.426
Post-operative complications	Yes	3	0	0.253
	No	16	13	
Bar Removed	Yes	8	7	0.513
	No	11	6	

*CI* confidence interval

When we assessed patient satisfaction using the SSQ we found that those who visited the website tended to be more satisfied, even though this did not reach statistical significance (Table 3). All patients who visited the website said that they would have the operation again compared to 76.9 % of those who did not (*p* = 0.031). Despite having more complications those who visited the website were equally or more satisfied with the post-operative outcome when compared with the group that did not visit the website and had no post-operative complications.

**Table 3:** Patients’ satisfaction using the Brompton’s single step questionnaire (SSQ)

Variable	Value	Did not visit website (*n* = 13)	Visited website (*n* = 19)	*p*-value
Health in general after the operation	Much better now (5)	3 (23.1 %)	6 (31.6 %)	0.095
	Somewhat better (4)	4 (30.8 %)	11 (57.9 %)	
	About the same (3)	5 (38.5 %)	2 (10.5 %)	
	Somewhat worse (2)	1 (7.7 %)	0	
	Much worse now (1)	0	0	
	Median	3	4	
Exercise capacity after the operation	Much better now (5)	5 (38.5 %	12 (63.2 %)	0.249
	Somewhat better (4)	4 (30.8 %)	2 (10.5 %)	
	About the same (3)	2 (15.4 %)	4 (21.1 %)	
	Somewhat worse (2)	2 (15.4 %)	1 (5.3 %)	
	Much worse now (1)	0	0	
	Median	4	5	
Extent the chest looks interfere with the pre-operative social activity	Extremely (5)	7 (53.8 %)	7 (36.8 %)	0.505
	Quite a bit (4)	4 (30.8 %)	9 (47.4 %)	
	Moderate (3)	0	2 (10.5 %)	
	Slightly (2)	2 (15.4 %)	1 (5.3 %)	
	Not at all (1)	0	0	
	Median	5	4	
Extent the chest looks interfere with post-operative social activity	Not at all (5)	7 (53.8 %)	12 (63.2 %)	0.6
	Slightly (4)	4 (30.8 %)	5 (26.3 %)	
	Moderately (3)	1 (7.7 %)	0	
	Quite a bit (2)	0	1 (5.3 %)	
	Extremely (1)	1 (7.7 %)	1 (5.3 %)	
	Median	5	5	
Satisfaction with overall post-operative appearance	Extremely satisfied (5)	3 (23.1 %)	7 (36.8 %)	0.628
	Very satisfied (4)	6 (46.2 %)	6 (31.6 %)	
	Satisfied (3)	3 (23.1 %)	5 (26.3 %)	
	Dissatisfied (2)	1 (7.7 %)	1 (5.3 %)	
	Very dissatisfied (1)	0	0	
	Median	4	4	
Bothered by the surgical scars	Not at all (5)	12 (92.3 %)	14 (73.7 %)	0.184
	Very slightly (4)	1 (7.7 %)	4 (21.1 %)	
	Slightly (3)	0	1 (5.3 %)	
	A little bit (2)	0	0	
	A lot (1)	0	0	
	Median	5	5	
Impact operation had on social life	Major Improvement (5)	5 (38.5 %)	5 (26.3 %)	0.553
	Improvement (4)	6 (46.2 %)	11 (57.9 %)	
	No change (3)	2 (15.4 %)	2 (10.5 %)	
	Worse now (2)	0	1 (5.3 %)	
	A lot worse now (1)	0	0	
	Median	4	4	
Pre-operative self esteem	1-10	3.7 (2.9–4.5)	4.4 (3.5–5.2)	0.280
	Median	3	4	
Post-operative self esteem	1–10	7.3 (5.9–8.7)	7.8 ( 7–8.6)	0.693
	Median	8	8	
Pain during hospital stay	None (5)	0	0	0.855
	Very mild (4)	2 (15.4 %)	7 (36.8 %)	
	Mild (3)	0	1 (5.3 %)	
	Moderate (2)	9 (69.2 %)	4 (21.1 %)	
	Severe (1)	2 (15.4 %)	7 (36.8 %)	
	Median	2	2	
Pain interfere with day to day activity now	Not at all (5)	10 (76.9 %)	9 (47.4 %)	0.138
	Very slightly (4)	1 (7.7 %)	5 (26.3 %)	
	Slightly (3)	2 (15.4 %)	5 (26.3 %)	
	A little bit (2)	0	0	
	A lot (1)	0	0	
	Median	5	4	
Pain now	No (5)	7 (53.8 %)	5 (26.3 %)	0.253
	Occasionally (4)	4 (30.8 %)	12 (63.2 %)	
	Mild- no painkillers (3)	2 (15.4 %)	1 (5.3 %)	
	Mild-painkillers (2)	0	1 (5.3 %)	
	A lot (1)	0	0	
	Median	5	4	
Conscious about metallic bar	Not at all (5)	1 (7.7 %)	5 (26.3 %)	0.154
	Slightly (4)	5 (38.5 %)	8 (42.1 %)	
	Moderately (3)	3 (23.1 %)	3 (15.8 %)	
	Quite a bit (2)	4 (30.8 %)	2 (10.5 %)	
	Extremely (1)	0	1 (5.3 %)	
	Median	3	4	
Overall satisfaction with the final result	Extremely satisfied (5)	6 (7.7 %)	8 (42.1 %)	0.762
	Very satisfied (4)	3 (23.1 %)	8 (42.1 %)	
	Satisfied (3)	3 (23.1 %)	3 (15.8 %)	
	Dissatisfied (2)	0	0	
	Very dissatisfied (1)	1 (7.7 %)	0	
	Median	4	4	
Chest looks difference	Major Improvement (5)	7 (53.8 %)	8 (42.1 %)	0.694
	Improved (4)	5 (38.5 %)	11 (57.9 %)	
	No change (3)	1 (7.7 %)	0	
	Worse now (2)	0	0	
	A lot worse now (1)	0	0	
	Median	5	4	
Going back, would you have the operation again?	Yes (10)	10 (76.9 %)	19 (100 %)	0.031
	Unsure (5)	2 (15.4 %)	0	
	No (0)	1 (7.7 %)	0	
	Median	10	10	
Overall obtainable score	0–95 (95 % CI)	60 (54–66)	62 (59–66)	0.65
	Median	62	62	

*CI* confidence interval

For the calculation of overall obtainable score: pre-operative self-esteem score was subtracted from the post-operative self-esteem score

## Discussion

Our results showed that the launch of a patient information website considerably increased the numbers of patients being referred and subsequently had an operation for repair of their pectus deformity. Nuss correction was introduced in our department in 2008 and it is now used for the treatment of PE patients. Prior to the launch of our website in 2010, only 1 patient had Nuss surgery in our thoracic surgery department. Over the last 5 year an average of 11 patients per year is having Nuss surgery for repair of PE. Our website had a considerable amount of hits per year leading to over a thousand contacts and 400 e-mail enquires underpinning the interest of the general public on pectus.

We decided to assess satisfaction of the PE patients who underwent Nuss correction of their deformity as we wanted to keep our group homogeneous and also use SSQ to assess satisfaction which is validated only for PE patients. When we sub-grouped our patients to those who visited the website prior to having surgery and those who did not, we found that both groups scored satisfactorily in SSQ. The Nuss operation had a positive psychological and physical impact on our patients and overall improved their quality of life. Krasopoulos et al. found similar outcomes post Nuss surgery using the SSQ [14] which were confirmed from later research groups [15–17].

The evidence for physical benefits though strong are unlikely to ever be supported by a randomised controlled trial. In a time of financial constraints it is easy for government or insurance funding bodies to see this type of surgery as a vulnerable target for savings [3]. Thus integrating patient reported outcomes as part of the clinical pathway as we have done in this study is especially important.

**Fig 2:**
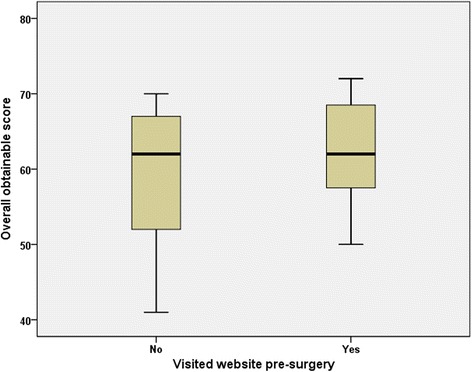
Total score obtained by the Bromptons’ single step questionnaire (SSQ) sub-grouped by patients who visited the pectus website prior to surgery and those who did not

The overall obtainable score calculated by SSQ was not different for our two subgroups (*P* = 0.65) but patients who did not visited the website had a greater spread of scores, from 41 to 70 with the former just reaching satisfactory levels. On the other hand patients who visited the website prior to surgery had scores ranging from 50 to 72 (Fig. 2). It is evident that well informed patients about what the surgery involves and what will happen after surgery tends to increase satisfaction with surgical outcomes. Despite having more complications patients who visited the website and obtained information about their surgery and their pathology were equally satisfied with the post-operative outcomes. Most of the patients are very young and active and we explain that we do not consider and treat pectus as a medical disease but as a variant of normal aiming to improve their chest cosmesis.

## Conclusions

By improving patients’ information about pectus surgery we improved their satisfaction after surgery. This might be because we managed their expectations and improved their recovery by providing better access to information. Collecting patient reported outcomes as part of the clinical pathway is essential in measuring success after surgery.

### Ethics approval and consent to participate

Data were anonymised therefore did not require ethics committee approval. Informed consent was obtained from participants prior to completion of the questionnaires.

## Abbreviations

PC: pectus carinatum
PE: pectus excavatum
SSQ: single step questionnaire

## References

[1] Sellke FW, del Nido PJ, Swanson SJ (2005). Sabiston and Spencer: Surgery of the chest.

[2] Zhao J, Luo L, Xiao L, Gu L, Sun T (2013). Psychological trauma of funnel chest in adolescents and the appropriate age for minimally invasive surgery repair. Clin Med J.

[3] NHS England. Clinical Commissioning Policy Proposition: Surgical correction for pectus deformity (all ages). Available at: https://www.engage.england.nhs.uk/consultation/clinical-commissioning-wave2/user_uploads/b10-x01-pectus-surgry-policy-prop.pdf. (Accessed 21 February 2016).

[4] Ji Y, Liu W, Chen S, Xu B, Tang Y, Wang X (2011). Assessment of psychosocial functioning and its risk factors in children with pectus excavatum. Health Qual Life Outcomes.

[5] Soares TR, Henriques-Coelho T, Vilaça J, Silva AR, Carvalho JL, Correia-Pinto J (2012). Quality of life evaluation of the patients and parents satisfaction after Nuss procedure in the management of Pectus Excavatum. Rev Port Cir Cardiotorac Vasc.

[6] Knudsen MV, Grosen K, Pilegaard HK, Laustsen S (2015). Surgical correction of pectus carinatum improves perceived body image, mental health and self-esteem. J Pediatr Surg.

[7] Brochhausen C, Turial S, Müller FK, Schmitt VH, Coerdt W, Wihlm JM (2012). Pectus excavatum: history, hypotheses and treatment options. Interact Cardiovasc Thorac Surg.

[8] Desmarais TJ, Keller MS (2013). Pectus carinatum. Curr Opin Pediatr.

[9] Rice RE (2006). Influences, usage, and outcomes of Internet health information searching: multivariate results from the Pew surveys. Int J Med Inf.

[10] Hansberry DR, Ramchand T, Patel S, Kraus C, Jung J, Agarwal N (2014). Are we failing to communicate? Internet-based patient education materials and radiation safety. Eur J Radiol.

[11] Wheeler R, Foote K (2000). Pectus excavatum: studiously ignored in the United Kingdom?. Arch Dis Child.

[12] Kelly RE (2008). Pectus excavatum: historical background, clinical picture, preoperative evaluation and criteria for operation. Semin Pediatr Surg.

[13] Heart of England NHS Foundation Trust. 2015. Pectus Surgery. [Online]. Available at: www.pectus.co.uk (Accessed 22 June 2015).

[14] Krasopoulos G, Dusmet M, Ladas G, Goldstraw P (2006). Nuss procedure improves the quality of life in young male adults with pectus excavatum deformity. Eur J Cardiothorac Surg.

[15] Hennig M, Kuebler JF, Petersen C, Metzelder ML (2012). General practitioners assessment highlights excellent patient satisfaction following bar removal after Nuss procedure in children and adolescents. Eur J Pediatr Surg.

[16] Jacobsen EB, Thastum M, Jeppesen JH, Pilegaard HK (2010). Health-related quality of life in children and adolescents undergoing surgery for pectus excavatum. Eur J Pediatr Surg.

[17] Metzelder ML, Kuebler JF, Leonhardt J, Ure BM, Petersen C (2007). Self and parental assessment after minimally invasive repair of pectus excavatum: lasting satisfaction after bar removal. Ann Thorac Surg.

